# Barriers and enablers to public access defibrillation – an international RAND-UCLA consensus study

**DOI:** 10.1186/s13049-026-01589-2

**Published:** 2026-03-13

**Authors:** K. C. Thies, C. Metelmann, B. Metelmann, T. Marks, T. Scquizzato, C. D. Deakin, R. Greif, E. Baldi, T. Barry, B. W. Böttiger, J. Bray, H. J. Busch, M. L. Caputo, S. Cheskes, R. Cresta, E. Degraeuwe, A. A. Doshi, M. M. Ekkel, D. Elschenbroich, D. Fredman, L. Gamberini, J. Ganter, F. L. Henriksen, C. J. Jagtenberg, M. Khalemsky, T. A. Kooy, K. G. Monsieurs, E. Moens, W. M. Ng, J. S. Pooth, S. Prasse, D. D. Salcido, A. Scapigliati, S. Schnaubelt, S. S. Scholz, P. Shahriari, P. Snobelen, R. Stieglis, B. Strickmann, H. L. Tan, G. Trummer, S. Vercammen, W. A. Wetsch, M. P. Müller

**Affiliations:** 1 Department of Anaesthesiology, Intensive Care, Emergency Medicine, Transfusion Medicine and Pain Therapy, University of Bielefeld, EvKB University Hospital of Bielefeld, Campus Bielefeld-Bethel, Burgsteig 13, 33167 Bielefeld, Germany; 2 Department of Anaesthesiology, Intensive Care, Emergency and Pain Medicine, University Medicine Greifswald, Greifswald, Germany; 3 Department of Anaesthesiology and Intensive Care Medicine, University Hospital Ulm, Ulm, Germany; 4 Department of Anesthesia and Intensive Care, IRCCS San Raffaele Scientific Institute, Milan, Italy; 5 University Hospital Southampton, Southampton UK & South Central Ambulance Service, Otterbourne, UK; 6 Faculty of Medicine, University of Bern, Bern, Switzerland; 7 Division of Cardiology, Fondazione IRCCS Policlinico San Matteo, Pavia, Italy; 8 Cardiac Arrest and Resuscitation Science Research Team (RESTART), Fondazione IRCCS Policlinico San Matteo, Pavia, Italy; 9 UCD School of Medicine, University College Dublin, Dublin, Ireland; 10 Faculty of Medicine, and University Hospital of Cologne, Department of Anaesthesiology and Intensive Care Medicine, University of Cologne, Cologne, Germany; 11 German Resuscitation Council (GRC), Ulm, Germany; 12 Monash School of Public Health and Preventive Medicine, Monash University, Melbourne, Australia; 13 Department of Emergency Medicine, Faculty of Medicine and Medical Center, University of Freiburg, Freiburg, Germany; 14 Cardiocentro Ticino Institute-Ente Ospedaliero Cantonale, Lugano, Switzerland; 15 Ticino Cuore Foundation, Lugano, Switzerland; 16 Faculty of Biomedical Sciences, Università Della Svizzera Italiana, Lugano, Switzerland; 17 Department of Emergency Medicine, Family and Community Medicine, Faculty of Medicine, University of Toronto, Toronto, ON Canada; 18 Ticino Canton EMS Federation, Bellinzona, Switzerland; 19 Department of Internal Medicine and Paediatrics, Ghent University, Ghent, Belgium; 20 Department of Pediatrics, AZ Sint-Lucas, Ghent, Belgium; 21 Department of Emergency Medicine, University of Pittsburgh, Pittsburgh, PA USA; 22 Amsterdam UMC Location AMC, Anesthesiology, Meibergdreef 9, Amsterdam, The Netherlands; 23 Charité – Universitätsmedizin Berlin, corporate member of Freie Universität Berlin and Humboldt-Universität Zu Berlin, Berlin, Germany; 24 Heartrunner Citizen Responder System, Heartrunner Sweden AB, Solna, Sweden; 25 Emergency Department, Maggiore Hospital Carlo Alberto Pizzardi, Bologna, Italy; 26 Department of Anaesthesiology and Critical Care, Faculty of Medicine, Medical Center – University of Freiburg, University of Freiburg, Freiburg, Germany; 27 Department of Cardiology, Odense University Hospital, Odense, Denmark; 28 School of Business and Economics, Vrije Universiteit Amsterdam, Amsterdam, the Netherlands; 29 Department of Management, Jerusalem Multidisciplinary College, Jerusalem, Israel; 30 Stan, Citizen Responder Network HartslagNu, Elburg, Netherlands; 31 Antwerp University Hospital and University of Antwerp, Edegem, Belgium; 32 Ghent University, Ghent, Belgium; 33 Department of Emergency Medicine, Ng Teng Fong General Hospital, Jurong East, Singapore; 34 Mobile Retter E.V, Köln, Germany; 35 Cardiac Anesthesia and Postoperative ICU, Fondazione Policlinico A.Gemelli, IRCCS. Institute of Anesthesia and Intensive Care, Catholic University of the Sacred Heart, Rome, Italy; 36 Italian Resuscitation Council, Bologna, Italy; 37 Emergency Medical Service Vienna, Vienna, Austria; 38 Department of Emergency Medicine, Medical University of Vienna, Vienna, Austria; 39 PULS - Austrian Cardiac Arrest Awareness Association, Vienna, Austria; 40 Emergency Medical Services, Capital Region of Denmark, Ballerup, Copenhagen, Denmark; 41 Peel Regional Paramedic Services, Mississauga, ON Canada; 42 Emergency Medical Service, District of Gütersloh (Kreis Gütersloh), Gütersloh, Germany; 43 Department of Clinical and Experimental Cardiology, Amsterdam UMC, University of Amsterdam, Amsterdam, the Netherlands; 44 Netherlands Heart Institute, Utrecht, the Netherlands; 45 Department of Cardiovascular Surgery, University Heart Center Freiburg - Bad Krozingen`, Medical Faculty – University of Freiburg, Freiburg, Germany; 46 EVapp NGO, Ghent, Belgium; 47 Department of Anaesthesiology, Intensive Care, and Emergency Medicine, St. Josefs Hospital Freiburg, Freiburg, Germany; 48 Institute of Emergency Care, Pedagogy of Health and Nursing Science, University of Pecs, Pecs, Hungary

**Keywords:** Automated external defibrillators, Out-of-hospital cardiac arrest, Cardiopulmonary resuscitation, Defibrillation, Emergency medical services, Public access defibrillation, Bystander intervention, Survival rate

## Abstract

**Background:**

Immediate cardiopulmonary resuscitation and early defibrillation are key determinants of survival after out-of-hospital cardiac arrest (OHCA). Public access defibrillation (PAD) remains inconsistently implemented, with major gaps in automated external defibrillator (AED) availability, integration and use. This study aimed to identify and prioritise barriers and enablers to PAD implementation and to highlight emerging deployment models for future research.

**Methods:**

During the third International Community First Responder Symposium (April 2024, Hinterzarten, Germany), 46 experts from 14 countries participated in a structured RAND–UCLA Appropriateness Method consensus study. In moderated discussions, participants identified barriers and enablers to PAD, which were grouped into four thematic fields: availability and accessibility, usability and awareness, technological and systemic aspects, and financial and maintenance concerns. Nine statements were formulated and rated on a 9-point Likert scale; strong consensus was defined a priori as a median ≥ 7 with ≥ 80% of ratings in the 7–9 range.

**Results:**

All nine statements met criteria for strong consensus. Key barriers included limited 24/7 AED access, poor coverage in residential areas, lack of centralised, real-time AED registries, insufficient public training and awareness, legal concerns for lay responders, patchy integration with emergency medical services, and device and maintenance costs. Key enablers comprised mandatory AED registration and live mapping, community training, legal protection for lay users, equipping police and fire services with AEDs within a “nearest vehicle” strategy, and improved data sharing between AEDs and hospitals. Experts also highlighted innovative deployment concepts, including use of postal and transport fleets, Vertical Take off and Landing drones, predictive positioning models and low-cost device designs.

**Conclusion:**

This international consensus study underscores the need for coordinated policy, robust AED registries, widespread training and multi-tier response models to improve PAD implementation. The identified priorities and innovative deployment strategies offer an agenda for future system development.

**Supplementary Information:**

The online version contains supplementary material available at 10.1186/s13049-026-01589-2.

## Background

Out-of-hospital cardiac arrest (OHCA) remains a major public health concern, with survival outcomes highly dependent on a variety of factors with early defibrillation representing a key variable. The presence of a shockable rhythm and the rapid availability and application of an automated external defibrillator (AED) significantly increase the likelihood of survival [1]. Within the first two minutes, over 75% of public OHCA cases present with a shockable rhythm, yet this prevalence declines rapidly with time [2]. Evidence shows that bystander defibrillation prior to emergency medical services (EMS) arrival leads to significantly improved survival rates [3] with hospital discharge rates of 53% when laypersons defibrillate compared to 23% when on-duty first responders perform defibrillation [4].

Despite strong evidence supporting early defibrillation, AED use remains inconsistent and suboptimal due to limited accessibility [5], lack of public awareness [6–8], and legal barriers [9]. In many cases, AEDs are unavailable where and when they are needed most, particularly in residential areas, where the majority of OHCA incidents occur [10]. Furthermore, AED integration into First Responder systems is often inadequate, with delays in accessing AEDs because of poor mapping, lack of real-time registry updates and functionality, as well as issues with emergency call taking and dispatch guidance [11–13].

Against this background, there is a need to better understand which system-level barriers and enablers most strongly affect public access defibrillation (PAD) and how they should be prioritised across different health systems. The aim of this study was to develop an international expert consensus on key barriers and enablers to PAD implementation and to identify practical system-level strategies to address them. These strategies, spanning AED deployment, organisational policies, the roles of first responders and laypersons, as well as emerging implementation concepts, are intended to inform policy, programme development and future research.

## Methods

We conducted a RAND–UCLA Appropriateness Method consensus study during a PAD symposium held in 2024 as part of the third International Community First Responder Conference in Hinterzarten, Germany. The conference’s objectives, participant composition, and additional outcomes have been described previously [14].

In summary, the symposium’s steering committee (MPM, BM, CM, KCT, RG, CDD, TS) invited participants from previous symposia [15, 16] and researchers with recent publications in the field, The invitation strategy strived to ensure diversity for geography, and genders as well as professional backgrounds by inviting experts from different fields (research, clinical, administrative and community sectors, see Supplement Table 1) [14]. A total of 46 experts from 14 countries across Asia, Australia, Europe, and North America participated and consented to the publication of the results. The Ethics Committee of the University of Greifswald granted a waiver for informed consent, as no patient data were involved.

Before the symposium commenced, all registered participants were invited to complete an anonymous, open ended online survey, listing up to three barriers that impeded delivery of an AED to OHCA patients before ambulance arrival. During the symposium, all experts (i.e. the full author group) took part in a moderated plenary discussion chaired by WAW and HLT, in which the survey results were presented and additional barriers and enablers were discussed. On this basis, the panel developed candidate consensus statements in open plenary: the moderators proposed draft formulations summarising areas of apparent broad agreement, participants suggested modifications, and wording was iteratively refined until no major objections remained. Issues raised only by a minority of participants or where views remained strongly divergent were not advanced as formal consensus statements.

The final statements were then rated using the RAND–UCLA Appropriateness Method (RAM) [17] with an anonymous, real-time audience-response system (PINGO, University of Paderborn, Germany). Each statement was displayed sequentially on a central screen and on participants’ mobile devices, read aloud by the moderator, and scored on a 9-point Likert scale (1–3 inappropriate, 4–6 uncertain, 7–9 appropriate) by all experts present. Approval was defined as a median > 6.5, and strong consensus was predefined as all ratings falling within the 7–9 range. Descriptive statistics were calculated using Microsoft Excel.

After the symposium, the steering committee reviewed the survey responses, discussion notes and voting results and, for analysis and reporting purposes, inductively organised the material into broader thematic fields. This thematic framework was developed post hoc and was not introduced before or during statement formulation or voting in order to avoid framing the discussion or biasing the content of the statements. Drafts of the manuscript were subsequently circulated to all panel members (i.e. the expert group) for review of accuracy and clarity; this stage was limited to clarifications and did not alter the content or results of the consensus statements.

## Results

Of the 46 experts, 27 (59%) completed the pre-symposium survey; all responses are summarised in Supplement Table 2. During the symposium, the full panel used these survey findings as a starting point for a moderated plenary discussion, in which they identified key barriers and enablers affecting PAD programmes.

### Key insights on the statements and level of consensus

The plenary discussion itself covered four thematic fields: availability and accessibility, usability and awareness, technological and systemic aspects, and financial and maintenance issues. The nine formal consensus statements reflect the first three fields and are summarised in Table 1.

**Table 1:** Summary of expert ratings of consensus statements on barriers and enablers to public access defibrillation (PAD)

***Thematic Field: AED Availability and Accessibility***				
**Statement**	**Median**	**Mean**	**SD**	**Consensus Level**
*We need more AEDs that are available 24/7*	9	8.4	1.17	Strong Consensus
*We need more AEDs that are available 24/7*	9	8.7	1.0	Strong Consensus
*AED should be available before ambulance arrival*	9	8.3	1.6	Strong Consensus
***Thematic Field: Usability and Awareness***				
**Statement**	**Median**	**Mean**	**SD**	**Consensus Level**
*The community should be trained in the use of an AED*	9	8.5	1.1	Strong Consensus
*AED registries should be available and constantly updated*	9	8.6	1.2	Strong Consensus
*AED delivery should not delay chest compressions*	9	8.3	1.25	Strong Consensus
***Thematic Field: Technological and Systemic Aspects:***				
**Statement**	**Median**	**Mean**	**SD**	**Consensus Level**
*AED data should be exported and made available for hospitals*	8	8.3	1.2	Strong Consensus
*AED registration should be mandatory*	9	8.3	1.1	Strong Consensus
*Police and fire service should carry AED and be dispatched to OHCA*	9	8.6	1.1	Strong Consensus

The table presents all consensus statements developed by the expert panel, grouped into three thematic fields: (1) AED availability and accessibility, (2) usability and awareness, and (3) technological and systemic aspects. Each statement was rated on a 9-point Likert scale (1–3 = disagreement, 4–6 = uncertainty, 7–9 = agreement) using the RAND–UCLA Appropriateness Method. For each statement, the median, mean and standard deviation (SD) of ratings are shown, together with the corresponding consensus level. Strong consensus was defined a priori as a median rating ≥ 7 with ≥ 80% of panel ratings in the 7–9 range; all statements listed in this table met these criteria

#### AED availability and accessibility

Based on the RAND–UCLA ratings, there was strong consensus on the need to increase the number of AEDs with unrestricted 24/7 access, particularly in locations with limited access outside business hours. AED availability before ambulance arrival was considered crucial for improving survival in OHCA, underscoring the importance of both strategic device placement and reliable round-the-clock access.

#### Usability and awareness

Experts emphasised the need for broad AED training that includes not only medical personnel but also non-medical staff and community members. A centralised, real-time AED registry was identified as an essential tool to support emergency call centres and coordinate responses. At the same time, participants agreed that AED delivery must not delay the initiation of CPR, reinforcing the importance of rapid but coordinated interventions and appropriate training for all responders.

#### Technological and systemic aspects

Data sharing between AEDs and hospitals was recognised as a valuable tool for enhancing post-resuscitation care and enabling data-driven debriefings. Mandatory AED registration at the point of purchase was strongly supported to ensure integration into emergency response networks and live mapping systems. There was also strong consensus on equipping police and fire vehicles with AEDs as a means of improving response rates and reducing time to shock. In settings with longer EMS response times, integrating AED-equipped first responders or AED-delivery drones was viewed as a promising strategy to further shorten time to defibrillation.

#### Financial and maintenance aspects

Experts reported that high purchase costs for AEDs, cabinets and installation limit deployment in public and residential areas and remain a major constraint on PAD implementation. They also noted that ongoing maintenance requirements, including regular checks and replacement of pads and batteries, add to long-term costs and may result in inactive or non-functional devices. Several participants emphasised the need for lower-cost, portable AED models and simplified maintenance arrangements to support sustainable expansion. However, these financial and maintenance issues are reported descriptively rather than as separate consensus statements because, although recognised as important, views differed on how far specific funding and maintenance models could be generalised and prioritised across health systems.

## Discussion

In this international consensus study at the Third International Community First Responder Symposium, 46 experts from 14 countries rated barriers and enablers to PAD across three thematic fields: availability and accessibility, usability and awareness, as well as technological and systemic factors.

The consensus highlighted inadequate AED availability in residential areas, low public confidence in AED use, and uneven integration of AEDs into emergency response systems as major, internationally shared problems. To derive practical guidance, we interpreted these findings in the context of recent PAD literature. This synthesis yielded the following system-level recommendations to improve AED accessibility and early defibrillation:

- Mandatory AED registration and real-time mapping
  Establish nationally or trans-regionally coordinated AED registries that are electronically linked to dispatch systems, allowing real-time mapping of device locations visible to call-takers and first responder apps. A mandatory AED registration upon purchase would help close gaps in coverage. The absence of comprehensive, up-to-date registries has been shown to hinder efficient AED dispatch and guidance [18]. Countries such as Denmark and Sweden already maintain nationwide AED registries integrated with emergency dispatch, enabling rapid location of accessible devices [19–21].
- Increased AED coverage in residential areas
  Prioritise AED placement in high-risk residential neighbourhoods, where most OHCAs occur but device density is low [10, 22]. Spatial analyses from Copenhagen and other cities show that optimised redistribution towards OHCA “hot spots”, particularly in residential zones, can substantially increase modelled bystander access [22]. These concerns mirror prior studies reporting locked or inaccessible AEDs after business hours, suboptimal placement relative to OHCA incidence and an overall shortage of devices in many communities [18, 23–29].
- Enhanced public awareness and training
  Expand public education, embedding AED (and CPR) training in schools, workplaces and community settings to increase confidence and willingness to act. Bystander defibrillation is associated with markedly higher survival [4], but fear of doing harm, legal uncertainty and perceived complexity remain key barriers [9, 30–32]. Previous work has also described ongoing debate about the balance between early defibrillation and continuous chest compressions, as well as training gaps, lack of confidence, device-related complexity and persistent misconceptions among lay responders and some professionals [33–37]. Countries that promote CPR/AED training through community and school programmes report higher survival, supporting these strategies [38, 39].
- Legal protections for lay responders
  Strengthen or introduce Good Samaritan provisions that explicitly allow lay people to use AEDs and protect them from liability when acting in good faith. Recent legislative changes in several European countries demonstrate that such laws can reduce perceived legal barriers and support broader public involvement [40, 41].
- Integration with first responder systems and AED apps
  Live AED registries, smartphone-activated first responder systems and dispatch protocols should be aligned so that rescuers are guided to both the victim and the nearest accessible AED in real time. Challenges with incomplete or non-integrated AED mapping, outdated or poorly synchronised registry data and suboptimal first responder deployment have been reported in several systems [42–45]. Systems such as SAUV Life, myResponder, Heartrunner, PulsePoint AED and FirstAED show that linking computer-aided dispatch with AED apps can increase early defibrillation in practice [19–21, 46–48].
- Multi-tier deployment and “nearest vehicle” strategies
  Equip police and fire service vehicles with AEDs and dispatch them alongside ambulances in suspected OHCA, following a “nearest vehicle” principle. Police and fire units often arrive before EMS, and police AED programmes have been shown to shorten time to first shock and improve survival from shockable rhythms compared with EMS alone [49, 50].
- Drone-assisted AED delivery in delayed-response areas
  In regions with prolonged EMS response times due to distance or traffic, AED-equipped drones can provide rapid delivery and reach patients faster than ground-based responders. Pilot projects in Sweden and other countries have shown that drones can arrive several minutes before the ambulance in a substantial proportion of dispatches, demonstrating feasibility and potential clinical benefit [51–53]. Further research and carefully designed pilot schemes are needed to address regulatory and operational challenges and to integrate drones as a complementary component of multi-tier PAD systems.
- Funding and Maintenance
  Financial and maintenance issues were identified as major barriers, with device, cabinet, installation and ongoing pad/battery costs limiting PAD expansion, especially for smaller organisations [54]. These constraints highlight the need for cheaper, more portable AED models [54–57]. Health technology assessment from Ireland suggests that PAD is only likely to be cost-effective when devices are targeted to selected high-yield locations, underscoring the need for centrally planned expansion [56]. In the UK, a community defibrillator fund and the centrally funded roll-out of AEDs to all state schools exemplify government–charity co-funding [58, 59]. Experience from national schemes suggests that sustainable PAD networks are often built on mixed funding models. In such models, a nationwide smartphone-activated citizen-responder system and AED registry are operated as a non-commercial partnership between EMS and a national foundation or public body. Individual devices, particularly in residential areas, are then co-financed by municipalities, local foundations and private organisations [60]. Against this background, our consensus suggests that combining central infrastructure support with locally shared device funding is a promising approach for scaling and maintaining PAD programmes.

### Emerging implementation strategies for future research

Modelling studies have suggested that using public points of interest as postal collection boxes as fixed AED locations could nearly double AED coverage in urban settings and improve access in residential neighbourhoods [61, 62]. This concept builds on this idea by integrating AED delivery into postal logistics, using scheduled routes and postal drones as a dual-use platform for both mail and time-critical medical devices. Such an approach has the potential not only to improve time to defibrillation, but also to address structural inequities in access to PAD in sparsely populated or geographically remote regions.

Another promising approach is to use Vertical Take-off and Landing drones (VTOL) which, because of their higher speed, can cover nearly ten times the area of standard multicopter drones and deliver AEDs while hovering above the OHCA scene. Operated as a shared resource between fire, police, and ambulance services and dispatched from a single control centre, such a VTOL-based system could achieve broader coverage at lower cost than a conventional multicopter fleet [53].

Cost-effective innovation efforts include open-source AED prototypes [63], low-cost mains-powered home devices, compact “single-use” handheld AEDs [64] and, more recently, smartphone-powered AED concepts [65], all aiming to reduce hardware and maintenance costs compared with conventional devices. These developments could lower the capital cost of PAD programmes and make wide-area deployment on shared VTOL- or postal-drone platforms more realistic.

Modelling studies of bus-mounted AEDs and “AED taxis” suggest that embedding defibrillators into transport fleets could substantially increase effective PAD coverage and reduce time to defibrillation compared with static devices alone [66, 67]. Extending this principle to other mobile assets, such as, ride-sharing fleets and service vans—may offer similar benefits, particularly in rural and remote settings where fixed AEDs and responder density are limited. By contrast, autonomous ground robots have mainly been proposed for urban last-mile/last-metre delivery; AED-specific “robotic ambulance” concepts describe robots transporting an AED to nearby OHCAs in smart-city/campus-type environments, while broader robotics literature supports the practicality of autonomous last-mile delivery platforms more generally [68–70].

Looking ahead, PAD systems are likely to become more adaptive and multi-modal. Spatiotemporal prediction and optimisation models could be used to dynamically position fixed and mobile AEDs according to anticipated OHCA risk [24, 62, 71–73]. while dispatch algorithms select the “best available asset” in real time – citizen responder, police or fire vehicle, VTOL drone or ground robot – to maximise the chance of an early shock [51, 74, 75]. In parallel, integrating AEDs into existing infrastructure such as parcel lockers, smart lampposts, EV-charging points and high-risk residential complexes could create a ubiquitous, low-maintenance AED network [76–78]. Together with frugal device innovations, these strategies illustrate how future PAD systems might blend predictive analytics, diverse carrier platforms and everyday urban infrastructure to bring an AED to the patient in time.

## Limitations

Despite the broad international representation of experts, research groups from well-resourced countries were particularly well represented. We were unable to identify subject-matter experts from several resource-limited regions, including parts of Eastern Europe, South America, and Africa. Although we also strived for diversity across professional sectors, certain stakeholder groups, for example public safety, urban planning, patient and survivor representatives, and community organisations, were under-represented. This limits the extent to which our findings capture the full spectrum of contextual, infrastructural, and community perspectives that influence PAD implementation.

Experts were purposively sampled based on prior work in PAD and first responder systems and are likely to be highly engaged “early adopters” of such programmes. This may introduce optimism bias, with feasibility and acceptability of some interventions potentially overestimated compared with jurisdictions with less established infrastructure or political support.

Our conclusions are based on a structured expert consensus process rather than empirical outcome data or a formal systematic review with evidence grading. The RAND–UCLA ratings summarise the perceived appropriateness and priority of selected strategies, informed by participants’ interpretation of the evidence and their local experience. However, they do not provide effect sizes or cost-effectiveness estimates. In addition, we focused on a limited number of key themes, so other relevant barriers and enablers may not be represented. All nine statements that were voted on met the predefined threshold for strong consensus. This should not be interpreted as implying that all aspects of PAD deployment are settled or uncontroversial.

The statements should be viewed as structured, experience-based priority-setting guidance that requires local adaptation to legal, organisational and financial conditions rather than as guideline-grade recommendations.

## Conclusion

This international RAND–UCLA consensus study highlights the need for a coordinated, system-level approach to public access defibrillation. Key areas include mandatory AED registration with real-time mapping, better coverage in residential areas, integration of AEDs into EMS and smartphone-activated first responder systems, and widespread CPR/AED training with legal protection for lay rescuers. Multi-tier response models that embed AEDs in police and fire services, alongside drone or fleet-based delivery as well as mixed funding and maintenance models, provide a pragmatic agenda for policy, implementation and research to improve survival after out-of-hospital cardiac arrest.

## Supplementary Information

Supplementary Material 1.

Supplementary Material 2.

## Data Availability

The data used generated and analysed during the current study are available from the corresponding author on reasonable request.

## Abbreviations

AED: Automated external defibrillator
CPR: Cardiopulmonary resuscitation
EMS: Emergency medical services
OHCA: Out-of-hospital cardiac arrest
PAD: Public access defibrillation
RAM: RAND/UCLA Appropriateness Method; RAND means Research ANd Development, and UCLA University of California Los Angeles.
VTOL: Vertical Take Off and Landing
