# Supplementary material for: Barriers and enablers to public access defibrillation – an international RAND-UCLA consensus study
Source: Scand J Trauma Resusc Emerg Med. 2026 Mar 13;34:66. doi: 10.1186/s13049-026-01589-2 (PMC13063452; doi:10.1186/s13049-026-01589-2)
Supplement: Supplementary file 1 — Supplementary Material 1. [file 13049_2026_1589_MOESM1_ESM.pdf]

		Number (%)
**Gender**	Female	11 (24%)
	Male	35 (76%)
**Country**	Australia	1 (2%)
	Austria	1 (2%)
	Belgium	4 (9%)
	Canada	2 (4%)
	Denmark	2 (4%)
	Germany	17 (37%)
	Ireland	1 (2%)
	Israel	1 (2%)
	Italy	4 (9%)
	Netherlands	5 (11%)
	Singapore	1 (2%)
	Sweden	2 (4%)
	Switzerland	3 (7%)
	United States of America	2 (4%)
**Professional Background**	Bio-science engineering	1 (2%)
	Business economist	2 (4%)
	Data manager	1 (2%)
	Health scientist	2 (4%)
	Mathematician	1 (2%)
	Nurse	2 (4%)
	Paramedic	3 (7%)
	Philosopher	1 (2%)
	Physician	33 (72%)
