# Supplementary material for: Barriers and enablers to public access defibrillation – an international RAND-UCLA consensus study
Source: Scand J Trauma Resusc Emerg Med. 2026 Mar 13;34:66. doi: 10.1186/s13049-026-01589-2 (PMC13063452; doi:10.1186/s13049-026-01589-2)
Supplement: Supplementary file 2 — Supplementary Material 2. [file 13049_2026_1589_MOESM2_ESM.pdf]

Supplement 2: Answers to the pre-symposium survey

Please list up to 3 barriers, which impede delivery of AED to OHCA before ambulance arrival.

Participant	Barrier 1	Barrier 2	Barrier 3
1	Lack of public access AEDs in strategic locations	Public awareness and willingness to use AEDs	Technological and logistical challenges in quickly pinpointing the nearest available AED
2	Distance to AED	lack of AED registration and registries	public awareness
3	Placement	inproper use	delay
4	Availabilty 24/7	number of AED	unknown location of AED
5	Distance	time from case to alert CFR	usability of app
6	Protocol	availability	quality
7	In	not visible at night-time or in fogged weather	no registration in an AED registre
8	AED not accessible 24/7	AED density too low	AED not in public register
9	No registry	not available 24/7	too few AEDs
10	AEDs Not registered	AED not publically available (locked behind doors)	not enough AEDs in rural areas
11	Availability/accessibility	inability to articulate location of PAD	wayfinding/signage of AED
12	Allocation of certain responders to picking up AED	availability 24/7	accurate register
13	Adequate mapping	24/7 availability	active guidance by first responder system to AED or not
14	Time between alerting EMS and other responders	infrastructure	lack of (knowledge of) AEDs
15	Culture	Money	Leading
16	Volunteers without (or with expired) CPR license aren't allowed to deliver one		
17	Not enough responders	not enough 24/7 AEDs	
18	Lack of knowledge and awareness of AEDs in the community		
19	accurate geolocation	unavailibility during specific hours/days	
20	AEDs not registered	locations without h24 access	unclear instructions for the FR
21	Location	distance	knowledge of use

22	Being unaware if the nearest AED location	low volunteers density	
23	24/7 acessability	residential areas	unknown locations
24	Accessibility	z axis	access to patient, AED numbers overall
25	Placed in suboptimal locations	locked in	
26	Different local organisation	poor technological implementation	low rate of community training
27	24/7 availible AED closed by	geolocation	how do I know that the AED comes if I am first with the CA-victim
